# A Guide to Carrying Out a Phylogenomic Target Sequence Capture Project

**DOI:** 10.3389/fgene.2019.01407

**Published:** 2020-02-21

**Authors:** Tobias Andermann, Maria Fernanda Torres Jiménez, Pável Matos-Maraví, Romina Batista, José L. Blanco-Pastor, A. Lovisa S. Gustafsson, Logan Kistler, Isabel M. Liberal, Bengt Oxelman, Christine D. Bacon, Alexandre Antonelli

**Affiliations:** ^1^ Department of Biological and Environmental Sciences, University of Gothenburg, Gothenburg, Sweden; ^2^ Gothenburg Global Biodiversity Centre, Gothenburg, Sweden; ^3^ Institute of Entomology, Biology Centre of the Czech Academy of Sciences, České Budějovice, Czechia; ^4^ Programa de Pós-Graduação em Genética, Conservação e Biologia Evolutiva, PPG GCBEv–Instituto Nacional de Pesquisas da Amazônia—INPA Campus II, Manaus, Brazil; ^5^ Coordenação de Zoologia, Museu Paraense Emílio Goeldi, Belém, Brazil; ^6^ INRAE, Centre Nouvelle-Aquitaine-Poitiers, Lusignan, France; ^7^ Natural History Museum, University of Oslo, Oslo, Norway; ^8^ Department of Anthropology, National Museum of Natural History, Smithsonian Institution, Washington, DC, United States; ^9^ Royal Botanic Gardens, Kew, Richmond-Surrey, United Kingdom

**Keywords:** anchored enrichment, bait, high throughput sequencing, Hyb-Seq, Illumina, NGS, molecular phylogenetics, probe

## Abstract

High-throughput DNA sequencing techniques enable time- and cost-effective sequencing of large portions of the genome. Instead of sequencing and annotating whole genomes, many phylogenetic studies focus sequencing effort on large sets of pre-selected loci, which further reduces costs and bioinformatic challenges while increasing coverage. One common approach that enriches loci before sequencing is often referred to as target sequence capture. This technique has been shown to be applicable to phylogenetic studies of greatly varying evolutionary depth. Moreover, it has proven to produce powerful, large multi-locus DNA sequence datasets suitable for phylogenetic analyses. However, target capture requires careful considerations, which may greatly affect the success of experiments. Here we provide a simple flowchart for designing phylogenomic target capture experiments. We discuss necessary decisions from the identification of target loci to the final bioinformatic processing of sequence data. We outline challenges and solutions related to the taxonomic scope, sample quality, and available genomic resources of target capture projects. We hope this review will serve as a useful roadmap for designing and carrying out successful phylogenetic target capture studies.

## Introduction

High throughput DNA sequencing technologies, coupled with advances in high-performance computing, have revolutionized molecular biology. These advances have particularly contributed to the field of evolutionary biology, leading it into the era of big data. This shift in data availability has improved our understanding of the Tree of Life, including extant (Hug et al., 2016) and extinct organisms (e.g., Green et al., 2010). While full genome sequences provide large and informative DNA datasets and are increasingly affordable to produce, they pose substantial bioinformatic challenges due to their size (data storage and computational infrastructure bottlenecks) and difficulties associated with genomic complexity. Further, assembling full genomes is often unnecessary for phylogenomic studies if the main goal is to retrieve an appropriate number of phylogenetically informative characters from several independent and single-copy genetic markers (Jones and Good, 2016). In those cases, it may be preferable to focus sequencing effort on a reduced set of genetic markers, instead of the complete genome.

Several genome-subsampling methods have been developed, which offer advantages over whole genome sequencing (WGS), mostly regarding costs and complexity (Davey et al., 2011). There exist non-targeted genome-subsampling methods such as those based on restriction enzymes (RAD-seq and related approaches; e.g., Miller et al., 2007; Baird et al., 2008; Elshire et al., 2011; Tarver et al., 2016). While these methods produce a reduced representation of the genome, the sequences produced are effectively randomly sampled across the genome, which poses several potential problems. For example, the orthology relationships among RAD-seq sequences are unknown, mutations on restriction sites generate missing data for some taxa, the odds of which increase with evolutionary time, and adjacent loci may be non-independent due to linkage disequilibrium (Rubin et al., 2012).

In contrast, the target capture method (Albert et al., 2007; Gnirke et al., 2009) offers a different genome-subsampling alternative. It consists of designing custom RNA bait sequences, which hybridize (bind) with the complementary DNA region of the processed sample. In a subsequent step, the DNA fragments that hybridized with bait sequences are captured, often amplified *via* PCR, and then sequenced. The design and selection of bait sets for a phylogenomic study is an important decision that needs to be considered with the organism group and research question in mind.

**Figure 1:**
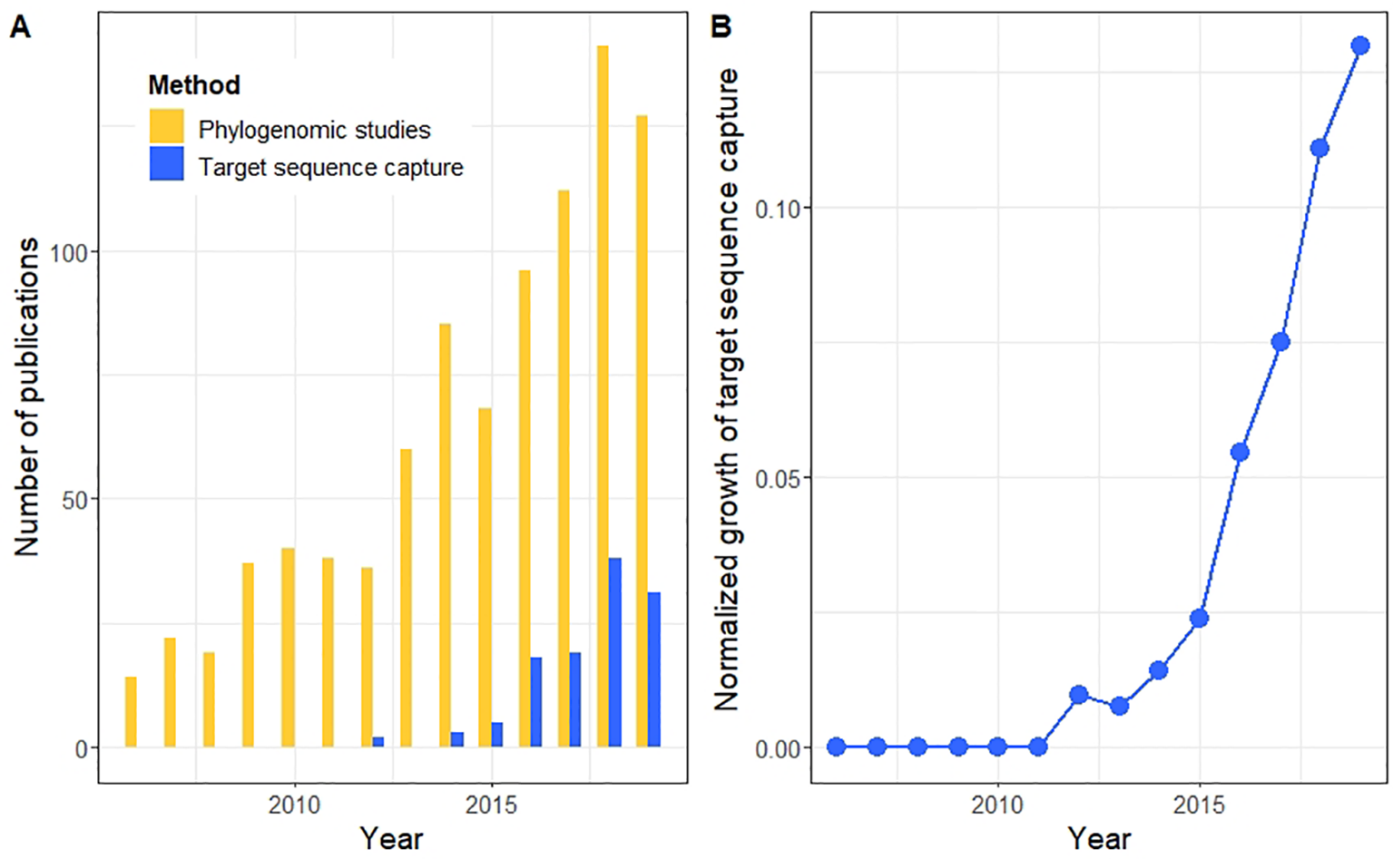
Published studies deposited in Web of Science that have used target sequence capture in phylogenetic research. **(A)** Number of publications by year (** our search included papers in Web of Science by December 20, 2019). **(B)** Normalized cumulative publications using target sequence capture in relation to other phylogenomic studies over time sorted by year of publication. We restricted our searches for studies published from 2006, the year of release of the first commercial high-throughput sequencer. We searched for Original Articles published in English in the category **‘**Evolutionary Biology**’**. We used eight combinations of keywords in independent searches that included the terms: **‘**hybrid**’** OR **‘**target*’ OR **‘**exon**’** OR **‘**anchored**’** AND **‘**enrichment**’** OR **‘**capture**’** AND **‘**phylogenom*’. We merged the datasets and we removed duplicated records by comparing unique DOIs (blue bars in panel A). These searches were contrasted with all other phylogenomic studies as specified by the keywords **‘**sequencing**’** AND **‘**phylogenom*’ (yellow bars in panel A).

Target capture focuses sequencing effort and coverage (also referred to as sequencing depth) on preselected regions of the genome. This allows for the targeted selection of large orthologous multi locus datasets, which is one of the reasons why target capture has been deemed the most suitable genome-reduction method for phylogenetic studies (Jones and Good, 2016), leading to its ever-growing popularity (**Figure 1**). Focusing the sequencing effort on a reduced number of loci also leads to higher coverage of these loci, compared to WGS. This feature also renders this method appropriate for museum specimen and herbarium samples, with possibly degraded DNA (Brewer et al., 2019), but see (Forrest et al., 2019). Deeper coverage at the loci of interest can further be essential for extracting Single Nucleotide Polymorphisms (SNPs) and for allele phasing. It leads to longer assembled targeted sequences (contigs), due to many overlapping reads in the targeted regions. This increased coverage at selected loci also allows pooling of more samples on fewer sequencing runs, thereby reducing costs.

**Figure 2:**
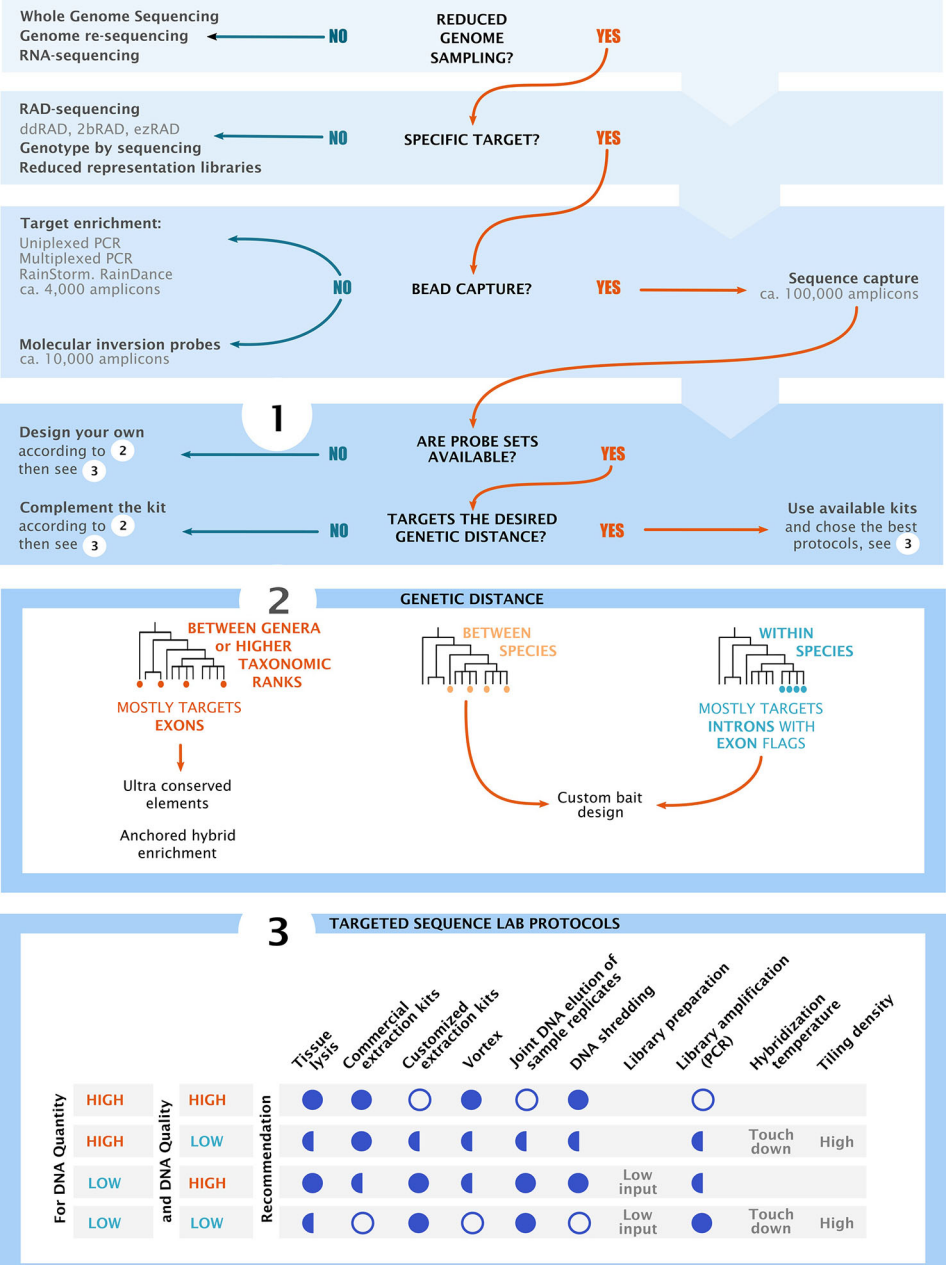
Decision chart and overview of the main considerations for project design in high throughput sequencing. The flow chart shows the most common groups of sequencing methodologies. Sections 1**–**3 summarize key components of project design, starting by choosing the sequencing methods, followed by bait design and finishing with the optimization of laboratory practices. Section 3 shows recommended (full circle), recommended in some cases (half circles) and not recommended (empty circles) practices based on input DNA quality and quantity. **“**Low input**”** refers to low input DNA extraction kits and **“**touch down**”** refers to temperature ramps at the hybridization and capture steps.

Every target sequence capture project is unique and requires a complex series of interrelated steps. Decisions made during data processing could have large effects on downstream analyses. Understanding the nature of data at hand, and the challenges of data processing, is crucial for choosing the most appropriate bioinformatic tools. Here, we present an overview and decision-making roadmap for target capture projects. We start at project design, then cover laboratory work (**Figure 2**), and finish with bioinformatic processing of target sequence capture data. This review does not attempt to capture all literature available in this topic; rather, it constitutes a summary of our own experiences from numerous target capture projects. It is particularly intended to help researchers and students new to the topic to design and carry out successful target capture experiments. Additional information can be found in other publications (e.g., Jones and Good, 2016; Dodsworth et al., 2019).

## Study Design

### Research Question

Developing a research question with testable hypotheses is an essential first step. Genomic data are sometimes generated without clearly defined goals, making it difficult to address specific questions *ad hoc*. One important early consideration is the taxonomic scope of the project, which influences taxon sampling, sequencing protocol and technology, and downstream data processing.

Some key questions to ask during target capture project design are:

- What is the intended phylogenetic scope of my study and how divergent should the selected loci be between my samples?
- Is a predesigned bait set available that satisfies the requirements I have for my target loci?
- What tissue material am I working with (e.g. fresh tissue or historical samples) and what is the expected quality and quantity of DNA I can extract?
- How can I optimize costs by pooling samples and using available sequence data for bait design, while ensuring sufficient sequencing coverage of all targeted loci?

Answering these questions will aid the choice of appropriate laboratory techniques and reduce technical issues in subsequent work (**Figure 2**). For example, using baits designed for organisms that are too divergent from the group of study will result in lower and less predictable capture rates. On the other hand, because designing custom baits can be expensive and because it is important to increase cross-comparability among studies, using a pre-designed bait set may be an attractive option for many target capture projects.

### Available Bait Sets

Generally, target capture baits are designed to align to target loci that are sufficiently conserved across the study group of organisms, to ensure unbiased capture that works equally well for all sequenced samples. At the same time these regions need to contain or be flanked by enough genetic variation that can inform about the phylogenetic relationships of these organisms. Therefore, the question of which baits to use and specifically whether to design a custom bait set or to use a predesigned bait set is an important consideration. The choice ultimately depends on how divergent the studied organism group is from the closest available bait set and how much genetic variation is needed in the target regions to resolve the phylogenetic question at hand. In order to make the best and most cost-efficient decision, it is important to have an overview of the available bait sets and of the common approaches used to design baits.

One common family of bait sets are those targeting highly conserved regions of the genome, such as Ultraconserved Elements (UCEs, Faircloth et al., 2012) or those produced by Anchored Hybrid Enrichment (Lemmon et al., 2012). These bait sets are designed by aligning several genomes between divergent sets of organisms, and identifying highly conserved regions (anchor-regions) that are flanked by more variable regions. These usually short regions are then selected for bait design. This approach has the advantage of recovering sets of loci that are highly conserved and thus can be applied to capture the same loci across divergent organism groups, while it also generally recovers part of the more variable and thus phylogenetically informative flanking regions (**Table 1**). On the other hand, due to their highly conserved nature, these regions are usually unsuitable to capture variation between populations, because of a limited number of variable sites on such shallow evolutionary scales at these loci. However even for these conserved loci several studies have recovered sufficient information to resolve shallow phylogenetic relationships below species level (e.g., Smith et al., 2014; Andermann et al., 2019).

**Table 1:** List of publicly available bait sets. This is not a complete list; it aims to highlight the taxonomic diversity of bait sets for broader organism groups. See the **Supplementary Table S1** for the number of baits in each set.

Name of bait set	Clade	Number of targeted loci	Reference
Arachnida 1.1Kv1	**Arthropoda:** Arachnida	1,120	Faircloth, 2017
Coleoptera 1.1Kv1	**Arthropoda:** Coleoptera	1,172	Faircloth, 2017
Diptera 2.7Kv1	**Arthropoda:** Diptera	2,711	Faircloth, 2017
Hemiptera 2.7Kv1	**Arthropoda:** Hemiptera	2,731	Faircloth, 2017
Hymenoptera 1.5Kv1 (hym‐v1)	**Arthropoda:** Hymenoptera	1,510	Faircloth et al., 2015
Hymenoptera 2.5Kv2 (hym‐v2)	**Arthropoda:** Hymenoptera	2,590	Branstetter et al., 2017
BUTTERFLY1.0	**Arthropoda:** Lepidoptera (Papilionoidea)	425	Espeland et al., 2018
BUTTERFLY2.0	**Arthropoda:** Lepidoptera (Papilionoidea: Hedylidae)	13*	Kawahara et al., 2018
Lepidoptera 1.3K-v1	**Arthropoda:** Lepidoptera	1,381	Faircloth, 2017
Actinopterygians 0.5Kv1	**Chordata:** Actinopterygii	500	Faircloth et al., 2013
Acanthomorphs 1Kv1	**Chordata:** Acanthomorpha	1,314	Alfaro et al., 2018
-	**Chordata:** Amphibia	8,706	McCartney-Melstad et al., 2016
-	**Chordata:** Anura	1,265	Portik et al., 2016
FrogCap	**Chordata:** Anura	~15,000	Hutter et al., 2019
AHE	**Chordata**	512	Lemmon et al., 2012
GENECODE	**Chordata:** Homo	205,031	Coffey et al., 2011
SqCL	**Chordata:** Squamata	5,312	Singhal et al., 2017
Coding Regions	**Chordata:** Squamata	3,888	Schott et al., 2017
Tetrapods-UCE-2.5Kv1/Tetrapods-UCE-5Kv1	**Chordata:** Tetrapoda	2,386	Faircloth et al., 2012
Anthozoa 1.7Kv1	**Cnidaria:** Anthozoa	1,791	Quattrini et al., 2018
Sphaerospira-Austrochlotitis-120-60-v2	**Mollusca:** Eupulmonata	2,648	Teasdale et al., 2016
Angiosperms-353	**Plantae:** Angiosperms	353*	Johnson et al., 2019
-	**Plantae:** Arecaceae	4,184	de la Harpe et al., 2019
PhyloPalm	**Plantae:** Arecaceae (Geonomateae)	795*	Loiseau et al., 2019
40916-Tapeworm	**Platyhelminthes:** Cyclophyllidea	3,641	Yuan et al., 2016
PenSeq	**Metagenomics:** Plant parasitic Oomycetes	~48*	Thilliez et al., 2019
MetCap	**Metagenomics:** Bacteria in soil samples	331 sequence clusters	Kushwaha et al., 2015
MEGaRICH	–	2,490	Noyes et al., 2017
ViroCap	**Virus**	Baits designed to identify viruses in human samples	Wylie et al., 2015

*Complete genes, including all exons. The target phylum is indicated in bold.

Another approach uses transcriptomic sequence data, often in combination with genomic sequence data, to identify exon loci that are sufficiently conserved across a narrower set of organisms (e.g., Bi et al., 2012; Hedtke et al., 2013; Ilves and López-Fernández, 2014). These bait sets are usually more taxon-specific compared to UCEs. Besides the conserved exon sequences, this approach recovers a larger part of the neighboring and more variable introns, leading to high numbers of phylogenetically-informative sites that are suitable for population-level questions (Gasc et al., 2016). Many studies choose to produce custom designed baits sets for specific organism groups (e.g. De Sousa et al., 2014; Heyduk et al., 2016; Couvreur et al., 2019), and many of these add to the pool of publicly available bait sets (**Table 1**).

### Designing Bait Sets

If there is no publicly available bait set that fits the organism group and research question of the planned experiment, researchers will have to design their own customized bait set. Bait development usually requires at least a draft genome or transcriptome reference, which may need to be sequenced *de novo* if not already available. To enable a high sensitivity when capturing target sequences, the designed baits should be sufficiently similar to these targets. For this reason, it is advantageous to choose a reference that is genetically similar to the study group, while ensuring that the resulting baits are not biased toward specific samples (Bragg et al., 2016). For example, it is recommendable to include at least one reference from the same genus if the aim is to sequence individuals of closely related species, or at least to include references of the same family when sequencing samples of related genera or higher taxonomic units.

Once genome or transcriptome references are produced or downloaded, the next step is selecting the target loci for bait design. Good starting points for identifying loci with the right amount of genetic variation are the bait design tools MarkerMiner 1.0 (Chamala et al., 2015), BaitFisher (Mayer et al., 2016) and MrBait (Chafin et al., 2018), as well as the simulation package CapSim (Cao et al., 2018). BaitFisher (with its filtering program BaitFilter) and MrBait allow for the design of baits targeting a broad taxonomic spectrum and different enrichment strategies. MrBaits uses multiple sequence alignments (MSAs) or genomes as inputs, while BaitFisher uses only MSAs. A more specific tool is MarkerMiner, which specializes in designing baits for single-copy nuclear genes in angiosperms using transcriptomes. Designed and selected bait sets can be tested in simulations using CapSim. Further useful methods are outlined in Faircloth (2017).

Besides sequence variation, another important consideration is to select loci without signs of paralogy, because baits designed from paralogous genes potentially capture multiple non-orthologous gene copies within a sample. Reconstructing evolutionary relationships between organisms based on a random mix of paralogous and orthologous gene copies will likely produce incongruent histories, leading to unrealistic scenarios of evolution (Doyle, 1987; Murat et al., 2017). Paralogy is an issue particularly for organisms where whole genome duplications have occurred, as is the case for many plants (Grover et al., 2012; Murat et al., 2017).

Having selected the target loci, multiple overlapping baits can be designed to cover these target regions, which is known as tiling (Bertone et al., 2006). This increases the chance of recovering several sequence fragments that cover the region of interest and ultimately increases the coverage. Having high coverage throughout the targeted region will be particularly important during the bioinformatic assembly of these sequences from the sequencing results, as explained in the Bioinformatics section of this review. The tiling density determines how much the bait sequences are overlapping and how many times a tile is laid over the gene region. Increasing tiling density is convenient for resolving regions in highly fragmented DNA as is the case of ancient DNA (Cruz-Dávalos et al., 2017), or when high sequence heterogeneity is expected within or between the samples.

Although most bait design relies on a reference or draft genomic sequence, some other approaches take advantage of other reduced-representation sequencing methods as a strategy to design target regions. For example, RADcap (Hoffberg et al., 2016) first utilizes a RAD-Seq approach (Sánchez Barreiro et al., 2017) in a subset of samples to discover genomic tracts and/or variable sites in a species lacking a genome assembly, and then relies on target capture to enrich for those regions in a wider set of samples. hyRAD (Suchan et al., 2016) takes a similar approach, although it circumvents RNA bait synthesis and instead physically transforms the ddRAD library molecules produced from a set of samples into biotinylated DNA baits for enrichment of other samples, including those with degraded DNA. Another approach, BaitSTR (Kistler et al., 2017), takes advantage of unassembled, medium-coverage short read genome sequencing data to discover and locally assemble massively parallel sets of short tandem repeats. These panels of thousands of short tandem repeats can then be captured for high-resolution population genomic inference.

### Baits for Prokaryotes

Target capture is also useful for the study of prokaryotes. Target capture is applied to the identification of the species composition in a community by designing baits for markers such as 16S or 18S rRNA genes (e.g., Cariou et al., 2018; Gasc and Peyret, 2018). The technique is also useful for the identification of prokaryote species based on their ecological function, by targeting gene families or functional DNA (e.g., Kushwaha et al., 2015; Noyes et al., 2017). For these applications, designing bait sets for prokaryotes involves similar considerations as those to be made for eukaryotes. The availability of reference sequences for bait design, the genetic distance between references and taxa (or gene family), and the taxonomic scope of the research question determine the capture specificity. Most prokaryote genomes fall below 5 Mb. Due to their small size, sequencing and assembling a prokaryote genome is connected to fewer financial and methodological hurdles compared to large eukaryote genomes. Moreover, there is an increasing number of reference sequences resulting from metagenomics experiments that can be used as references (Gasc et al., 2015).

Nevertheless, the biggest challenges of working with prokaryotes are related to the species concept in prokaryotes and the high frequency of horizontal gene transfer (see Gevers et al., 2005 for a detailed discussion). Two lineages of what is considered the same prokaryote species might differ in their gene variants and even gene presence. Thus, the bait sets for a prokaryote taxon must target ubiquitous and single-copy loci throughout known strains or gene families (Gevers et al., 2005; Gasc et al., 2015). Similarly, horizontal gene transfer between two taxa with no shared recent ancestry can result in the sequencing of two closely related gene copies from completely different lineages. This will result in the misidentification of lineages in a community and in erroneous phylogenetic reconstructions. Avoiding genes like those conferring antibiotic resistance or loci known to be transferred horizontally can reduce the confounding effects interfering with phylogenetic estimations (Gevers et al., 2005). Tools for designing bait sets targeting prokaryotes include HiSpOD (Dugat-Bony et al., 2011), KASpOD (Parisot et al., 2012), and Metabolic Design (Terrat et al., 2010).

## Laboratory Work

### DNA Extraction and Quantification

DNA extraction determines the success of any target capture experiment and requires special attention. Different protocols optimize either quality or scalability to overcome the bottlenecks posed by sample number, total processing time of each protocol, and input DNA quantity (Rohland et al., 2010; Schiebelhut et al., 2017). Purity and quantity of DNA yield varies depending on the protocol, taxon, and tissue. Old samples from museums, fossils, and tissues rich in secondary chemicals, such as in certain plants and archaeological tissues, are particularly challenging (Hart et al., 2016). But, in general, target capture sequencing can deal with lower quantity and quality of DNA compared to other methods such as WGS (Templeton et al., 2013; Blaimer et al, 2016) or RADseq in some cases (Harvey et al., 2016).

Commercially available DNA extraction kits use silica columns and may be ideal for large sets of samples while maintaining the quality of the yield. For instance, Qiagen^®^, Thermo Fisher Scientific and New England BioLabs produce a wide range of kits specialized in animal and plant tissues, and microbial samples. Their protocols are straightforward if the starting material is abundant and of high quality. The downsides of these kits are the high costs and, in few cases, they potentially produce low or degraded yield (Ivanova et al., 2006; Schiebelhut et al., 2017). However, modifications to the binding chemistry and other steps in column-based protocols can improve the recovery of ultra-short DNA fragments from difficult tissues such as ancient bone (Dabney and Meyer, 2019) and plant tissues (Wales and Kistler, 2019).

Customized extraction protocols can be less expensive and generally produce higher yield and purity, as research laboratories optimize steps according to the challenges imposed by their DNA material. These protocols are better at dealing with challenging samples but are more time-consuming. Examples include the cetyl trimethylammonium bromide (CTAB) protocol (Doyle, 1987) and adaptations thereof, which produce large yield from small tissues (Schiebelhut et al., 2017). CTAB-based protocols are particularly recommended for plant samples rich in polysaccharides and polyphenols (Healey et al., 2014; Schiebelhut et al., 2017; Saeidi et al., 2018). However, in historic and ancient samples, CTAB methods are occasionally deemed non-optimal and recent experiments have favored other methods (but see Brewer et al., 2019). The other methods recommended are a modified PTB (N-phenacyl thiazolium bromide) and column-based method for herbarium tissues (Gutaker et al., 2017), a custom SDS-based (sodium dodecyl sulfate) method for diverse plant tissues (Wales and Kistler, 2019), and an EDTA and Proteinase K-based method for animal tissues (Dabney and Meyer, 2019). All of these protocols optimized for degraded DNA extraction rely on silica columns with modified binding chemistry to retain ultra-short fragments typical in ancient tissues (Dabney et al., 2013).

Another protocol similarly aimed at extracting DNA from low-quality samples is the Chelex (Bio-Rad Laboratories, CA, USA) method, which is easy, fast and results in concentrated DNA. The downsides of using Chelex for DNA extractions are that the resulting single-strand DNA tends to be unstable for long-term storage (Hajibabaei et al., 2005) and that the protocol performs poorly with museum specimens (Ivanova et al., 2006). However, a modified Chelex protocol where the heating step is removed, results in more stable double-strand DNA (Casquet et al., 2012; Lienhard and Schäffer, 2019).

The curation of a historical or ancient sample determines the success of its DNA extraction (Burrell et al., 2015). A non-visibly-destructive extraction approach is best if the initial material is limited or impossible to replace (Garrigos et al., 2013), or for bulk samples (such as for insects) where all species may not be known *a priori* and morphological studies could be beneficial afterward (Matos-Maraví et al., 2019). However, yields from these minimally invasive methods are typically low, and better suited to PCR-based methods than genomic methods. If material destruction is unavoidable, it is best to use the tissue that is most likely to yield sufficient DNA. For instance, hard tissues like bones may be preferable to soft tissues that have been more exposed to damage (Wandeler et al., 2007). In animals, the petrous bone has emerged as a premium DNA source because it is extremely dense and not vascularized, offering little opportunity for chemical exchange and DNA loss. Moreover, DNA from ancient material should not be vortexed excessively or handled roughly during process to prevent further degradation (see Burrell et al., 2015 and Gamba et al., 2016 for extended reviews). General aspects of ancient DNA extraction are that A) an excess of starting material can decrease the yield and increase contaminants (Rohland et al., 2010); B) additional cleaning and precipitation steps are useful to reduce contaminants in the sample but also increase the loss of final DNA (Healey et al., 2014); and C) extraction replicates pooled before binding the DNA can increase the final yield (Saeidi et al., 2018). Current tissue-specific protocols for degraded and ancient DNA are compiled by Dabney and Meyer (2019).

Quantity and quality checks should be done using electrophoresis, spectrophotometry and/or fluorometry. Fluorometry methods like Qubit™ (Thermo Fisher Scientific) measure DNA concentration, even at very low ranges, and selectively measures DNA, RNA or proteins. Spectrophotometric methods like Nanodrop™ (Thermo Fisher Scientific) measure concentration and the ratio between DNA and contaminants based on absorbance peaks. If the ratio of absorbance at 260 nm and 280 nm is far from 1.8–2, it usually means that the sample contains proteins, RNA, polysaccharides and/or polyphenols that may inhibit subsequent library preparations (Lessard, 2013; Healey et al., 2014). Peaks between 230 and 270 nm are indications of DNA oxidation. Nanodrop™ provides precise and accurate measures within a concentration range from 30 to 500 ng/μL, but attention should be paid to solution homogeneity, delay time, and loading sample volume (Yu et al., 2017). Gel electrophoresis or automatized electrophoresis using TapeStation™ (Agilent Technologies) or the more sensitive Bioanalyzer™ (Agilent Technologies) systems measure fragment size distributions, DNA concentration, and integrity. Measuring DNA quantity is key before library preparation, capture (before and after pooling), and sequencing, to ensure an adequate input (Healey et al., 2014). Measuring protein contamination or the presence of inhibiting molecules present in the DNA sample is necessary before library preparation, as additional cleaning steps may be required.

### Library Preparation

A DNA sequencing library represents the collection of DNA fragments from a particular sample or a pool of samples, modified with synthetic oligonucleotides to interface with the sequencing instrument. Library preparation strategies compatible with Illumina sequencing involves fragmentation of the input DNA (shearing) to a specific size range that varies depending on the platform to sequence, adapter ligation, size selection, amplification, target capture or hybridization, and quantification steps. Most kits available require between 10 ng and 1000 ng of high-quality genomic DNA, but kits designed for low DNA input are becoming available, such as the NxSeq^®^ UltraLow Library kit (0.05 ng, Lucigen^®^) and the Illumina^®^ High-Sensitivity DNA Library Preparation Kit (as low as 0.2 ng, Illumina). As a general rule, high concentrations of starting material require less amplification and thus the library will have more unique reads (Rubin et al., 2012; Head et al., 2014; Robin et al., 2016). A minimum input of 1 µg microgram for library preparation is recommended when possible (Folk et al., 2015). It is possible to use lower input DNA amounts with every kit and still perform library preparation, but initial tests are advised (Hart et al., 2016). Ancient and degraded DNA requires modifications to these standard protocols. For example, shearing and size selection are usually not advisable because the DNA is already highly fragmented, and purification methods suitable for short fragments must be used. The 1 µg threshold is almost never attainable with ancient DNA, but custom library preparation strategy can still work with down to 0.1 ng of DNA with appropriate modifications (Meyer and Kircher, 2010; Carøe et al., 2018). Moreover, ligation biases inherent to most kit methods are especially pronounced at low concentrations, so these lab-developed methods are usually preferable for difficult DNA sources (Seguin-Orlando et al., 2013).

All short-read sequencing protocols require shredding high-molecular-weight genomic DNA into small fragments. The DNA is broken at random points to produce overlapping fragments that are sequenced numerous times depending on their concentration in the genomic and post-capture DNA. Covaris^®^ instruments are commonly used to fragment the DNA to a preferred size range using a sonication approach. Other methods use fragmentase enzymes, beads inserted directly into the biological sample, or ultrasonic water-baths. The fragment size of the library should be suitable for the sequencing chemistry and library preparation protocol. A target peak of 400 base pairs, for example, is adequate for second generation sequencing technologies like Illumina. For third generation sequencing technologies like PacBio^®^ or Oxford Nanopore Technologies, a peak of 5–9 kb may be adequate, but much larger fragments can also be accommodated (Targeted Sequencing & Phasing on the PacBio RS II, 2015). Degraded material from museum and ancient samples seldom requires any sonication, as mentioned above. After shearing the fragmented DNA is quantified to ensure adequate DNA concentration and size. If necessary, it can be concentrated on a speed vacuum or diluted in EB buffer or RNAse-free water, although drying samples can further damage degraded material. Miscoding lesions in chemically damaged DNA—e.g. from deaminated, oxidized, or formalin-fixed DNA—can be partially repaired using enzymes before library preparation (e.g. Briggs et al., 2009).

After shearing the ends of the fragmented DNA need to be repaired and adapters ligated to them. Depending on the library preparation protocol, adapters are ligated to either blunt-ends (both DNA strands end on the same nucleotide position) or an AT-overhang (one strand has extra A or T nucleotides). These adapters constitute complex oligonucleotides, containing the binding region for the polymerase for PCR amplification while also enabling sequencing by synthesis cycles on Illumina machines. Further the adapters contain the binding sites for the fragment to bind to the sequencing platform’s flow cell. Finally the adapters contain specific index sequences (barcodes), which are used to mark and distinguish all samples that are being processed.

It is important to pay close attention to the concentration of adapters applied to the DNA fragments. Lower adapter concentrations can reduce unwanted adapter dimers but result in biases against fragments with 5′-dT when using AT-overhang ligation (Seguin-Orlando et al., 2013). The bias is particularly problematic for historical and degraded samples, whose DNA fragments are naturally rich in overhang ends (Meyer et al., 2012).

There are different approaches how to assign specific adapter indeces to the fragments of specific samples. The indexing can be single (only one adapter contains index sequence) or dual (two adapters with two different index sequences). If the number of libraries in a single sequencing run is less than 48, using single indexing is enough. Dual indexing is necessary if more than 48 libraries need to be uniquely identified. Moreover, dual indexing reduces possible false assignment of a read to a sample (Kircher et al., 2012). Further, index swapping and the resulting false sample-assignment of sequences is a known problem of Illumina sequencing that can be minimized using dual-indexing (Costello et al., 2018). Adapters with their index sequence are ligated to both ends of the DNA fragment. After adapter ligation, a cleaning step with successive ethanol washes is carried out to remove the excess of reagents.

The next step is size selection (if necessary). Each sequencing platform has limits on the range of fragment sizes it is optimized for (see the *Sequencing* section). Fragments above or below those size thresholds may have reduced chances of binding to the flow cell surface, ultimately, reducing sequencing accuracy (Head et al., 2014). Therefore it is important to select fragments of the correct size range before before sequencing. Size selection can be done by recovering the target size band from an agarose gel or, more commonly, by using carboxyl-coated magnetic beads. In this step, the distribution of fragment length is narrowed and thus, the length of the targets that will be captured is optimized. Size selection must be done carefully to avoid DNA loss, especially if the DNA input is lower than 50 ng and degraded (Abcam^®^ - High Sensitivity DNA Library Preparation Kit Protocol V2). Size selection is not always necessary if the fragments already fall within the desired size range, or when any DNA loss would be detrimental (e.g. for historical and degraded samples). At the end of size selection, the size distribution of the selected fragments should be accurately measured using a Bioanalyzer system, or using a TapeStation system or an agarose gel.

### Target Capture

Capture takes place either in a solid-phase (or array) with baits bound to a glass slide (Okou et al., 2007), or using a solution-phase with baits attached to beads suspended in a solution (Gnirke et al., 2009). The latter has been shown to be more efficient (Mamanova et al., 2010; Paijmans et al., 2016), and because of workflow efficiency and handling, solid-phase capture has fallen out of favor in recent years. Capture protocols require between 100 and 500 ng of genomic library, although these bounds may be modified, for example, when low DNA content is expected (Perry et al., 2010; Kistler et al., 2017). During capture, pooled libraries are denatured and hybridized to RNA or DNA baits, which typically contain a biotin molecule. Then magnetic beads are added, which are coated in Streptavidin, which acts as a receptor to the biotin molecules. This leads to the baits, which are hybridized to the target DNA fragments, to bind to the magnetic beads. Using a magnet, these beads are then immobilized and the non-target fragments which are still in solution are washed off and discarded. After a purification step, post-capture PCR amplification is necessary to achieve a library molarity of the captured fragments sufficient for sequencing.

Assuming perfect input material, capture sensitivity and specificity depends on the similarity between bait and target, the length of the target, the hybridization temperature, and chemical composition of the hybridization reaction. To ensure the best capture conditions, it is important to closely follow the lab-instructions provided by the company that synthesized the baits, independently of using self-designed or commercial capture kits.

Baits have greater affinity and sensitivity increases the more similar the target sequence is to the bait sequence, thus sequence variation in the target sequence among samples can lead to differences and biases in capture efficiency across samples. Moreover, longer targets require bait tilling. Another common problem is low specificity when part of the target sequence hybridizes with other non-homologous sequence fragments, which can be the case when the target sequence contains repetitive regions or is affected by paralogy (i.e. several copies of the targeted area exist across the genome).

Adding blocking oligonucleotides can reduce the nonspecific hybridization of repetitive elements, adapters and barcodes (McCartney-Melstad et al., 2016). Blocking oligonucleotides are designed to bind a template at the same complementary region as an adapter or primer, thus blocking the amplification or hybridization of that particular fragment (Vestheim et al., 2011). As the blocking oligos target repetitive sequences, known non-targeted, or known contaminant sequences, their use results in the preferential amplification of targeted sequences. By reducing non-targeted binding, adding blocking oligos can increase capture specificity.

In the presence of few differences between bait and target sequences, baits can still capture less similar fragments at low hybridization temperatures. However, capture sensitivity decreases at higher temperatures as specificity between bait and target sequences increases, establishing different priorities and approaches for working with fresh or ancient DNA (Li et al., 2013; Paijmans et al., 2016). For example, for ancient DNA—where hybridization of contaminant sequences is likely—higher temperatures increase specificity toward non-contaminant DNA, but at the cost of capturing fewer fragments (McCormack et al., 2016; Paijmans et al., 2016). However, using a touch-down temperature array provides a good tradeoff between specificity and efficiency (Li et al., 2013; McCartney-Melstad et al., 2016). Arrays to capture regions of ancient and fragmented DNA reduce the hybridization to contaminant sequences without compromising hybridization to targets. Lower salt concentrations during hybridization also increase specificity, favoring the most stable bonds (Schildkraut and Lifson, 1965). Finally, Gasc et al. (2016) present a summary of methods for modern and ancient data, and Cruz-Dávalos et al. (2017) provide recommendations on bait design and tiling, both useful for ancient DNA.

### Amplification

An amplification step enriches the selected target fragments and is especially relevant for low input libraries, as DNA yield is proportional to the number of PCR cycles. However, PCR is the primary source of base substitution errors during library preparation, and too many PCR cycles can lead to a high percentage of PCR duplicates, which can preclude sequencing all loci with sufficient coverage, as some regions will be overrepresented. Aird et al. (2011) and Thermes (2014) review the causes of bias and propose modifications to reduce it. Their recommendations include extending the denaturation step, reducing the number of cycles if DNA input is high, and optimizing thermocycling. Although PCR-free library preparation workflows exist, for example to reduce identical reads for shotgun sequencing, they are not appropriate for capture-based experiments, and tend to result in extremely low yields. Around six PCR cycles of amplification pre-hybridization, and around 14–18 cycles after hybridization are recommended for an optimal capture efficiency and complexity of captured libraries (Mamanova et al., 2010).

Pooling takes place amongst prepared libraries to reduce costs and take advantage of sequencing capacity. Pooling libraries consists of assigning unique barcodes to a sample, developing libraries and pooling equimolar amounts of each library in a single tube, from which the combined libraries are sequenced. Indexes are selected so that the nucleotide composition across them is balanced during sequencing, and various protocols provide advice on index selection (Meyer and Kircher, 2010; Faircloth et al., 2012; Glenn et al., 2019). Balancing the index sequences is particularly crucial when very few libraries are sequenced in the same lane or a specific library dominates the lane.

Pooling samples before library preparations, also called “pool-seq”, can be used for projects with hundreds of samples and if tracing back individual samples is not relevant for the research question at hand (Himmelbach et al., 2014; Anand et al., 2016). This strategy is useful for the identification of variable regions between populations, especially when population sampling must be higher than what the budget allows for sequencing as individual libraries (Neethiraj et al., 2017). Because with this method it is possible to sample many individuals within a population, there is more information for detecting rare variants across the population. However, the design of the pool-seq strategy must be done carefully and must be congruent with the project: never pool together individuals or populations across which the project aims to find differences. For a more in-depth discussion on pool-seq strategies and protocols, see (Meyer and Kircher, 2010; Rohland and Reich, 2012; Schlötterer et al., 2014; Cao and Sun, 2016; Glenn et al., 2019).

### Determining Coverage

For target capture sequencing, coverage refers to the number of reads covering a nucleotide position in the target sequence. The desired coverage of the targeted loci dictates the choice of the sequencing platform and the number of libraries per lane. It is estimated from the sum length of all haploid targeted regions (G), read length (L), and number of reads produced by the sequencing platform (N) (Illumina coverage calculator, 2014). To calculate the coverage of a HiSeq sequencing experiment that produces 2 million reads (N), assuming paired-end reads (2x) of 100 bp length (L) and a total length (G) of 20 Mbp of targeted sequences, coverage will be:

$$Coverage=\frac{L\times N}{G}=\frac{\left(2\times 100\right)\times 2,000,000}{20,000,000\;bp}=20x$$

This calculation can assist in deciding optimal pooling strategies. For example, if 50x coverage is required for 20 Mbp of sequencing data, the sequencing platform must produce at least 5 million reads to achieve the desired coverage across the complete target. The same calculation can be used to calculate if and how many libraries can be pooled in a sequencing experiment. For example, if one is considering pooling three samples to produce paired-end reads of 100 bp length and a cumulative target region of 20 Mbp, every sample would receive an average coverage of 20/3 = 6.7. This might not be sufficient coverage for some downstream applications of the data.

It is important to keep in mind that the expected coverage is not always the resulting coverage when bioinformatically processing the sequencing data after sequencing. The final coverage depends on the GC nucleotide content of the reads, the quality of the library, capture efficiency, and the percentage of good quality reads mapping to the targeted region. For target capture specifically, the mean coverage of any target will vary depending on the heterozygosity, number of paralogous copies on the genome, and whether the target has copies in organelle genomes (e.g. mitochondria or chloroplasts), either of which would lead to capturing the wrong fragments, which ultimately will affect the coverage of the target sequences (Grover et al., 2012). It is not recommended to target both nuclear and organelle regions in a single bait design, because the high number of organelle copies per cell in an organism ultimately results in very low coverage for the nuclear targets.

### Sequencing

Sequencing platforms either carry out repeated clonal amplification of the provided DNA molecules or they only sequence or a single DNA molecule. Clonal amplification produces relatively short reads between 150 and 400 bp (Illumina^®^ and Ion Torrent^™^ from Life Technologies Corporation), while single molecule sequencing produces reads longer than 1 Kbp and as long as >1 Mbp (Oxford Nanopore Technologies and Pacific Biosciences). Capture approaches usually target relatively short fragments (ca. 500 bp), thus short-read methods are more efficient. However, improvements in the hybridization protocol are making the sequencing of captured fragments around 2 Kbp feasible, encouraging the use of long-read platforms in combination with target capture with the potential of increasing the completeness of the targeted region. For example, Bethune et al. (2019) integrated target capture using a custom bait set, and sequencing using MinION^®^ (Oxford Nanopore Technologies), to produce long portions of the chloroplast; their method was successful for silica-dried and fresh material of grasses and palms. Similarly, (Chen et al., 2018) designed a bait set from a frog DNA sample to recover targets from another two frog mitogenomes, then sequenced the targets using an Ion Torrent^™^ Personal Genome Machine^™^. Finally, Karamitros and Magiorkinis (2018) generated baits to target two loci in *Escherichia* Phage lambda and *Escherichia coli* and sequenced them with MinION^®^ (Oxford Nanopore Technologies), with a capture specificity and sensitivity higher than 90%.

Depending on the chosen sequencing method, many different types of reads can be generated. For Illumina sequencing, single-end and paired-end are the most commonly used reads. Single-end reads result from fragments sequenced in only one direction and paired-end reads from fragments sequenced in both the forward and reverse directions. Paired-end reads can have lower false identification rates if the fragment is short enough for redundant nucleotide calls using both directions, unlike single-paired (Zhang et al., 2016). Paired-end reads are also recommended for projects using degraded and ancient samples to improve base-calling where chemical damage is likely (Burrell et al., 2015), although short (75 bp) single reads can also provide an efficient sequencing option in those cases.

## Bioinformatics

### Data Storage and Backup

High-throughput sequencing produces large volumes of data, in the size range of at least tens to hundreds of Gigabytes (GB), which need to be stored efficiently. It is therefore important to plan for sufficient storage capacity for processing and backing up genomic data. In addition to the raw sequencing data, target capture projects typically generate a high volume of data that exceed the size of the original data 3- to 5-fold during the processing steps. This is due to several bioinformatic processing steps (outlined below), which produce intermediate files of considerable size for each sample. Assuming an average raw sequencing file size of 1–2 GB per sample, we recommend reserving a storage space of up to 10 GB per sample. Most importantly, the raw sequencing files should be properly backed up and preferably immediately stored on an online database such as the NCBI sequence read archive (Leinonen et al., 2011b, https://www.ncbi.nlm.nih.gov/sra) or the European nucleotide archive (Leinonen et al., 2011a, https://www.ebi.ac.uk/ena), which have an embargo option, preventing others to access the sequence data prior to publication. There may be additional national, institutional, or funding agency requirements concerning data storage, with the goal of increasing research transparency and reproducibility.

### Analytical Pipelines

The bioinformatic processing of target capture data, or high throughput sequencing data in general, is a broad field with many available tools and programs. Particularly for scientists without specific training in bioinformatics, this field can appear intimidating and difficult to navigate. To help guiding through the most essential steps, several pipelines have been developed, some specifically for target capture data or multilocus data more generally. Pipelines also differ in terms of which part of the post-sequencing workflow they cover. Some pipelines are particularly focused on the specific steps of assembly and recovery of longer compound sequences from the read data (sequence engineering), such as aTRAM (Allen et al., 2017) and HYBPIPER (Johnson et al., 2016). Other pipelines are more focused on guiding users through the complete process from cleaning raw sequencing reads to producing data structures that can be readily used for phylogenetic inference (e.g. Multiple Sequence Alignments or SNP datasets), such as PHYLUCE (Faircloth, 2016) or SECAPR (Andermann et al., 2018) (**Table 2**).

**Table 2:** Popular short read processing pipelines. Full circles stand for ‘Applies’, half circles for ‘Partly applies’, and empty circles for ‘Does not apply’ for the respective category of the pipeline.

	Read cleaning	Sequence engineering	Intron recovery	MSA generation	Allele phasing	SNP extraction	Ease of installation
aTRAM (Allen et al., 2017)	○	●	●	○	○	○	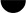
HYBPIPER (Johnson et al., 2016)	○	●	●	○	○	○	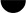
PHYLUCE (Faircloth, 2016)	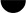	○	○	●	●	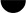	●
SECAPR (Andermann et al., 2018)	●	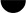	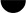	●	●	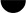	●

The choice of which pipeline to choose is usually based on the type of data at hand and on the intended use of these data. For example PHYLUCE is particularly streamlined for sequence data of UCEs and enables easy and fast processing of raw reads into MSAs. SECAPR on the other hand is designed for more general use as it combines the user-friendly design of PHYLUCE with additional steps of sequence engineering, making it more suitable for target capture data of any type. If the aim is to retrieve very long sequences including intron sequences flanking the targeted exons, HYBPIPER is the appropriate tool, yet it requires more bioinformatic knowledge to prepare the input data and to process the resulting sequences into data structures for phylogenetic analyses. Similarly, aTRAM enables generation of long sequences, using an iterative assembly approach, which we explain in more detail below.

All pipelines named above are usually used for short read data (Illumina). While our experience with processing long read data (i.e. Nanopore or PacBio) for target capture is limited, it appears that for these datatypes the use of standardized pipelines is not as common as for short read data. The lack of an established pipeline for long-read target capture is perhaps due to greater differences between datasets produced with these methods, in terms of coverage, fragment length, and intended purpose of the experiments. Nanopore sequencing is commonly used for assembling small genomes (e.g., Loman et al., 2015; Bethune et al., 2019) and while some workflows have been published as software packages, such as Nanopolish (based on Loman et al., 2015), the majority of studies using this sequencing technology apply a combination of bioinformatic toolbox commands to create project-specific workflows. Similarly, studies that have used PacBio sequencing in combination with target capture enrichment commonly create their individual customized workflows (e.g., Wang et al., 2015; Lagarde et al., 2017).

### Cleaning, Trimming, and Quality Checking

The first step after receiving and backing up raw read files is the removal of low-quality reads, of adapter contamination, and of PCR duplicates. These are usually done in conjunction, using software such as Cutadapt (Martin, 2011) or Trimmomatic (Bolger et al., 2014).

Low quality reads: Illumina reads are stored in FASTQ file format, which in addition to the sequence information contains a quality (PHRED) score for each position in the read, representing the certainty of the nucleotide call for the respective position. This information enables cleaning software to remove reads with overall low quality and to trim parts of reads below a given quality threshold.

Adapter contamination: Adapter contamination particularly occurs if very short fragments were sequenced (shorter than the read length). Adapter trimming software can usually identify adapter contamination, since the sequences of common Illumina adapters are known and can be matched against the read data to identify which sequences originate from these adapters. However, there can be problems in identifying adapter contamination if the adapter-originated sequences are too short for reliable detection. This problem is usually mitigated in paired-end data, where the overlap of read pairs can be used to identify adapter-originated sequences more reliably (Bolger et al., 2014).

Removing PCR duplicates: An additional recommended step is the removal of PCR duplicates, which are identical copies of sequences that carry no additional information and convolute further processing steps. This can be done using software such as the SAMtools function *markdup* (Li et al., 2009).

Finally, it is important to compile quality statistics for cleaned samples to determine if there are remaining biases or contamination in the data. FASTQC (Andrews, 2010), for example, calculates and plots summary statistics per sample, including the quality per read position, the identification of overrepresented sequences (possibly adapter contamination), and possible quality biases introduced by the sequencing machine. It is strongly recommended for all read files to pass the quality tests executed by FASTQC (or equivalent functions in some processing pipelines) before continuing to downstream data processing.

### Assembly of Reads Into Sequences

There are different avenues to proceed from the cleaned reads. The choice of which of these approaches to take depends mainly on the availability of a reference genome or reference sequences for the specific study-group and the intended final sequence product (e.g. consensus sequence alignments, allele sequences, SNPs). In one approach the raw reads are mapped to reference sequences (reference assembly), which enables the extraction of variable sites or the assembly of full sequences from overlapping read information. In another approach the raw reads are assembled into contigs (*de novo* assembly) which are collapsed into longer sequences. This is particularly useful if no reference sequences are available for the sequenced organisms. Yet another approach combines the two previous ones by first *de novo* assembling reads into contigs and then mapping reads against these contigs to recover allelic variation that is otherwise lost when collapsing reads into contigs.

Reference-based assembly: There are several mapping software packages that allow mapping (aligning) reads against a reference library. Commonly used read mapping software packages are the Burrows Wheeler Aligner BWA, (Li and Durbin, 2010), Bowtie (Langmead et al., 2009), and Minimap (Li, 2016). The resulting reference-assembly product is usually stored in BAM format. The reference-assembly approach collects the complete read variation for each locus and enables the user to extract SNPs, to phase reads belonging to different alleles or to simply build consensus sequences from the read variation. When mapping reads against a reference library (collection of reference sequences), the user must choose similarity thresholds, based on how similar the sequence reads are expected to match the reference sequence. The reference library can consist of a collection of individual reference sequences for the targeted loci (exons or genes) or of a complete reference genome (chromosomes), but see the consideration at the end of this paragraph. The aim of read mapping is to extract all sequence reads that are orthologous to a given reference sequence, while at the same time avoiding reads from paralogous genomic regions. A compromise must be made between allowing for sufficient sequence variation to capture all orthologous reads, while being conservative enough to avoid mapping reads from other parts of the genome. The choice of sensible similarity thresholds thus depends strongly on the origin of the reference library and the amount of expected sequence divergence between the reference sequences and the sequenced samples. It is usually of advantage to use a reference library consisting of all genetic regions with available sequence information, including loci that were not targeted during target capture, since these additional regions can act as filters that bind reads not desired in the dataset of enriched regions.

*De novo* assembly: Few non-model organisms have suitable (closely related) reference sequences available for reference-based assembly. To generate longer sequences from short read data, a common first step in those cases is *de novo* assembly. During *de novo* assembly, reads with sequence overlap are assembled into continuously growing clusters of reads (contigs) which are then collapsed into a single contig consensus sequence for each cluster. There are different *de novo* assembly software packages, which differ in their specific target use (short or long DNA or RNA contigs). Some of the commonly used software packages for assembling target capture data are ABySS (Simpson et al., 2009), Trinity (Grabherr et al., 2011), Velvet (Zerbino and Birney, 2008), and Spades (Bankevich et al., 2012). *De novo* assemblies are usually computationally very time intensive and generate large numbers of contig consensus sequences, only some of which represent the targeted loci.

In order to extract and annotate the contig sequences that represent targeted loci, a common approach is to run a BLAST search between the contig sequences on the one hand and the bait sequences or some other collection of reference sequences on the other hand (e.g., Faircloth et al., 2015). The above mentioned short read pipelines aTRAM, HYBPIPER, PHYLUCE, and SECAPR all contain functions that employ some BLAST algorithm to match the assembled contigs with reference sequences of the desired target loci. Further, there are computational tools such as Exonerate (Slater and Birney, 2005) for splicing and aligning coding nucleotide sequences on the assembled contigs, after matching these to a known locus.

Sometimes *de novo* and reference-assembly approaches are used in conjunction, where *de novo* assembly is used to generate a reference library from the read data for subsequent reference-based assembly (Andermann et al., 2019). The question arises, why not to directly use the bait sequences (more specifically: the reference sequences used for bait design) instead of the assembled contigs as reference library? Using the annotated contigs instead of the bait sequences as references has the advantage that these sequences are on average longer, since they usually contain sequences trailing the genomic areas that were captured (e.g. they may contain parts of intron sequences for exon-capture data). Another advantage is that this approach produces taxon-specific reference libraries, while the bait sequences, in most cases, are sampled from genetically more distant taxa. Another common question is why not using the contig sequences for downstream analyses, skipping the reference-based assembly altogether? In fact, contig sequences are commonly used for phylogenetic inference, yet depending on the assembly approach that was chosen, these sequences might be chimeric, consisting of sequence bits of different alleles. This property may bias the phylogenetic inference, as discussed in Andermann et al. (2019a). The combined approach will also enable the extraction of heterozygosity information as discussed below, which is usually lost when collapsing reads into contig sequences during *de novo* assembly.

Yet another promising path for *de novo* generation of even longer sequences from short read data are reference-guided *de novo* assembly pipelines, such as implemented in aTRAM. In this iterative approach, clusters of reads are identified that align to a given reference (e.g. the bait sequences) and are then assembled *de novo*, separately within each read cluster (locus). This process is repeated, using the resulting consensus contig sequence for each locus as reference for identifying alignable reads, leading to growing numbers of reads assigned to each locus, as reference sequences become increasingly longer in each iteration.

All following steps describe downstream considerations in case of reference-assembly data. If one decides to work with the contig data instead and omit reference-assembly, the contig sequences are ready to be aligned into MSAs and require no further processing.

### Assessing Assembly Results

**Figure 3:**
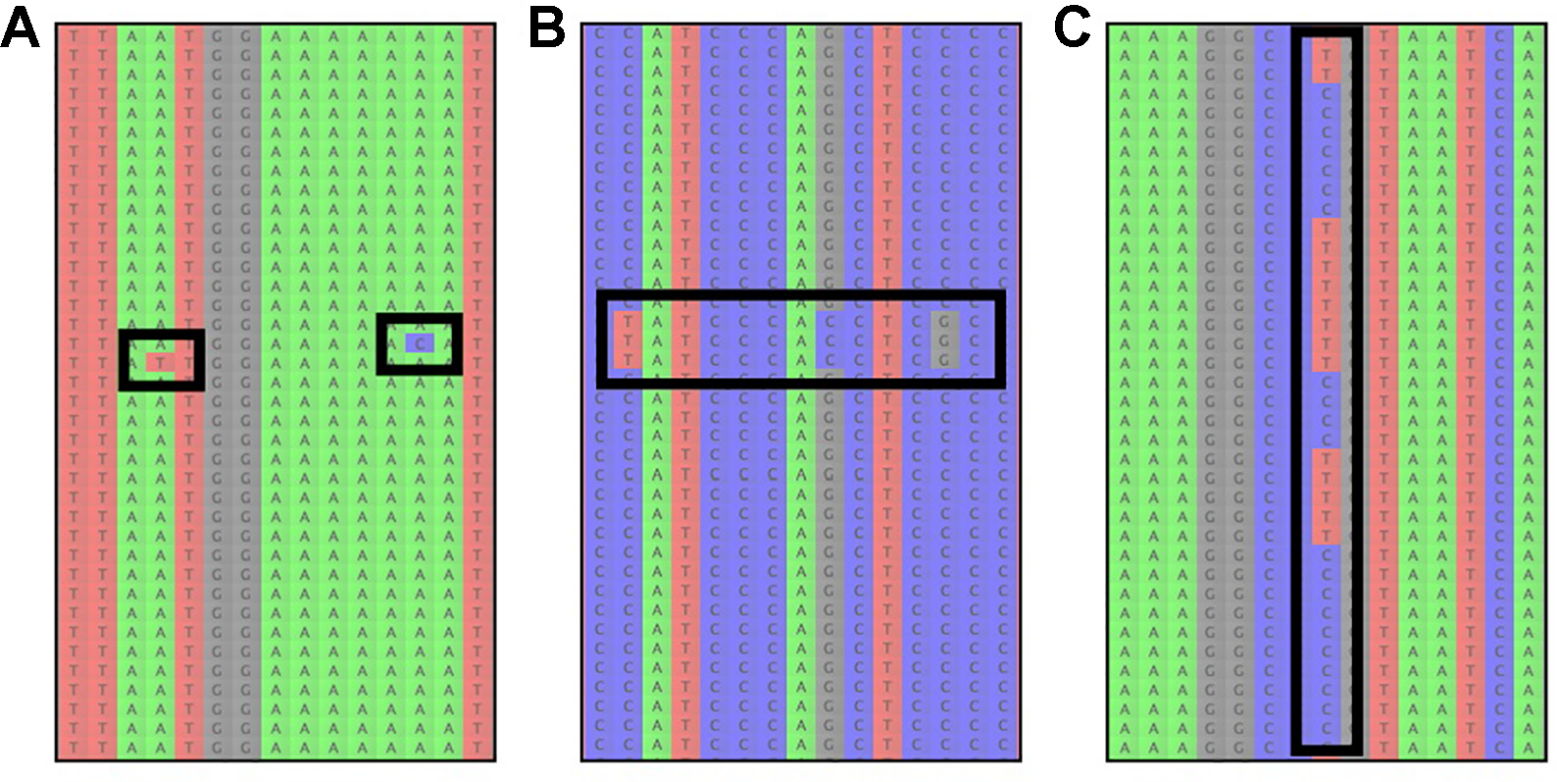
The most common sources of read-variation within reference-based assemblies of a given organism. **(A)** Sequencing errors are identifiable as single variants that are only present on an individual read and are generally not shared across several reads. **(B)** Paralogous reads are visible as blocks of reads with several variants shared among a low frequency of reads. Paralogous reads originate from a different part of the genome and are a result of gene or genome duplication. **(C)** Allelic variation can usually be identified by variants that are shared among many reads, occurring at a read frequency of approximately 1/ploidy-level, i.e. 0.5 for diploid organisms.

To evaluate reference-based assembly results, it is advisable to manually inspect some of the resulting read-assemblies and check if there are A) an unusual number of read errors (resulting from low quality reads) or B) signs of paralog contamination (incorrectly mapped reads; **Figure 3**). Read errors are identifiable as variants at different positions in the assembly, which only occur in individual reads (**Figure 3A**). If many reads containing read errors are found, it is recommendable to return to the read-cleaning step and choose a higher read-quality threshold, to avoid sequence reads with possibly incorrect low quality base-calls. Paralogous reads, on the other hand, are usually identifiable as reads containing several variants, which occur multiple times in the assembly (**Figure 3B**). However, a similar pattern is expected due to allelic variation at a given locus for diploid and polyploid samples (**Figure 3C**, Andermann et al., 2019).

These two scenarios (paralogous reads vs. allelic variation) can usually be distinguished by the amount of sequence variation between reads: alleles at a locus are not expected to be highly divergent for most taxa, with some exceptions (Thompson et al., 2014), while paralogous reads are expected to show larger sequence divergence from the other reads in the assembly. However, this is only true for paralogous reads stemming from loci that duplicated prior to the divergence of the study group (outparalogs, *sensu* Sonnhammer and Koonin, 2002). If instead gene- or partial genome-duplications occurred for lineages within the study group (inparalogs), these are usually not detectable through sequence variation alone. Instead, one can assess if paralogous reads are present by checking if reads stemming from more than N haplotypes are found in the assembly for an N-ploid organism, which happens when reads from different alleles and paralogous reads end up in the same assembly (e.g., Andermann et al., 2018). Additionally, the frequencies at which variants occur among the reads can assist in understanding if the reads stem from paralogous contamination or allelic variation. In the latter case, the frequency is expected to be 1/ploidy, while paralogous reads can occur at any frequency, depending on the copy number of the respective locus in the genome and depending on the sequence divergence from the targeted locus, which affects the capture efficiency. If paralogous reads are identified, it is recommended to exclude the effected loci from downstream analyses.

A different and more general measure of read-mapping success is assessing the read coverage. This simply constitutes an average of how many reads support each position of the reference sequence and therefore provides an estimate of how confidently each variant is supported. Read-coverage is an important measure for the subsequent steps of extracting sequence information from the reference assembly results and can be easily calculated with programs such as the SAMtools function *depth* (Li et al., 2009). In case of target capture it is generally advisable to aim for an average read coverage of at least 10 reads for a given locus for diploid organisms. This value is to be understood as a rule of thumb recommendation, which is based on the reason that it likely leads to multiple reads covering each haplotype at a given site (assuming diploid organisms), enabling allele phasing or SNP extraction at the site. If the read coverage is substantially lower than that, it is usually an indicator that either the enrichment process with the used baits did not work properly for the given locus (perhaps because the locus sequence in the sequenced individual is to divergent from the bait sequence), or it could be a result of the processing pipeline and the chosen reference sequence or mapping thresholds. In many cases the recovered read-coverage at many loci can be improved by testing different mapping and sequence similarity threshold settings for the specific dataset (see Andermann et al., 2018). However, if the sequencing experiment did not work as expected and coverage of all loci is low, it may still be possible to produce consensus sequences from loci with read coverage thresholds of at least 3, yet in these cases it will not be possible to extract allelic information (see Andermann et al., 2018). There are however statistical models for calculating genotype likelihoods from putative allelic variation in low coverage data (e.g. ancient DNA), implemented in the software ANGSD (Korneliussen et al., 2014).

### Extracting Sequences from Assembly Results

With all target reads assembled, there are different ways of compiling the sequence data for downstream phylogenetic analyses. One possible approach is to compile full sequences for each locus in the reference library by extracting the best-supported base-call at each position across all reads (e.g. the unphased SECAPR approach, see Andermann et al., 2018). This approach yields one consensus sequence for each given locus. Alternatively, to forcing a definite base-call at each position, those positions with multiple base-calls originating from allelic variation can be coded with IUPAC ambiguity characters (e.g., Andermann et al., 2019). In the latter case, it is important to check if the phylogenetic software that is used to analyze the resulting sequences can read these ambiguity characters, as some programs treat these characters as missing information.

Another approach is to separate reads belonging to different alleles through allele phasing (He et al., 2010; Andermann et al., 2019). Subsequently, a separate sequence can be compiled for each allele, yielding N sequences per locus for an N-ploid individual. However, no general software solutions for allele phasing of more than two alleles have been established for short-read data at this point (but see Rothfels et al., 2017, for long read solutions), which presents a major bottleneck for many studies working with polyploid organisms.

A third approach is the extraction of SNPs from the reference assembly results. In this case, only variable positions within a taxon group are extracted for each sample. SNP datasets are commonly used in population genomic studies, since they contain condensed phylogenetic information, compared to full sequence data. Even though large SNP datasets for population genomic studies are commonly produced with the RAD-seq genome subsampling approach, target capture produces data that can also be very useful for this purpose, as it usually provides thousands of unlinked genetic markers at high coverage that are present in all samples. This renders the extraction of genetically unlinked SNPs—a requirement for many downstream SNP applications—simple and straightforward (e.g., Andermann et al., 2019). Even though most phylogenetic methods are sequence based, some methods can estimate tree topology and relative divergence times using only SNPs instead (e.g., SNAPP, Bryant et al., 2012).

## Conclusions

There have been several initiatives to generate whole genome sequences of large taxon groups, such as the Bird 10,000 Genomes (B10K) Project, the Vertebrate Genomes Project (VGP), and the 10,000 Plant Genomes Project (10KP). While we share the enthusiasm surrounding the vision of ultimately producing whole genome sequences for all species, there is also substantial concern about the environmental impact of such large sequencing efforts (e.g., Philippe, 2011). Therefore we think that target sequence capture is likely to continue playing a substantial role, particularly in phylogenetic studies, also for the following reasons. Firstly, a substantial portion of all species are only known from a few specimens in natural history collections, often collected long ago or are too precious to use large amounts of tissue for sequencing to ensure the extraction of enough genomic DNA (as is required for the production of whole genomes). Secondly, sequencing costs for full genomes of many samples are still prohibitively high for research groups in developing countries, even though sequencing costs are rapidly decreasing. Thirdly, the complexity of assembling and annotating full genomes, especially using short-fragment sequencing approaches, is still a major bottleneck and requires suitable references among closely related taxa, which is lacking in many cases.

Other initiatives that are sequencing large groups of organisms with standardized target capture kits, such as the Plant and Fungi Tree of Life (PAFTOL, https://www.kew.org/science/our-science/projects/plant-and-fungal-trees-of-life) constitute a promising alternative to the mentioned full genome initiatives. To further accelerate the use of target capture we advocate A) sequencing and annotation of high-quality reference genomes across a wider representation of the Tree of Life, B) the establishment of data quality and processing standards to increase comparability among studies, such as those put forward by the computational pipelines mentioned in this review, and C) the availability of published bait-sets and target capture datasets on shared public platforms.

## Author Contributions

TA and MT authored the drafts of the paper. TA, MT, and PM-M prepared figures and conducted a review of the background literature. TA, MT, PM-M, RB, JB-P, AG, LK, IL, BO, CB, and AA reviewed drafts of the paper and contributed to individual sections of the manuscript. All authors approved the final version of the manuscript. We further note that the two first authors TA and MT contributed equally to this work. The order of their names in the author list was decided by coin toss.

## Funding

MT and CB were funded by the Swedish Research Council (2017-04980). PM-M was funded by the European Union’s Horizon 2020 Programme under the Marie Skłodowska-Curie (MARIPOSAS-704035) and the PPLZ program of the Czech Academy of Sciences (grant L200961951). RB received a Doctoral Fellowship and a Fellowship for Internship abroad from Coordination for the Improvement of Higher Education Personnel (CAPES, Processo 99999.000566/2015-02). TA and AA were funded by a Wallenberg Academy Fellowship. JB-P was funded by the European Union’s Seventh Framework Programme under a Marie Skłodowska-Curie fellowship (AlfalfaEvolution, Grant Agreement n. 625308). AA was further funded by the Swedish Research Council (B0569601), the Swedish Foundation for Strategic Research, and the Royal botanic Gardens, Kew. The funders had no role in study design, data collection and analysis, decision to publish, or preparation of the manuscript.

## Conflict of Interest

The authors declare no conflicts of interest. In particular, we do not have any relation to any of the companies or their products listed here, and the opinions expressed in this review are based on our own experiences and interpretations.

## Supplementary Material

The Supplementary Table S1 containing additional information regarding the publicly available bait sets can be found at: https://www.frontiersin.org/articles/10.3389/fgene.2019.01407/full#supplementary-material

Table S1 Supplementary information for available bait sets.

## Glossary

Bait sequences: Short RNA sequences (sometimes referred to as probes) that are synthesized to be complementary to target regions on the genome, which they are intended to bind to. The synthesized RNA strands are usually marked with the molecule biotin, which can bind to specific biotin receptors, e.g. streptavidin, which is located on the surface of magnetic beads.
Capture efficiency: Measure of the level of enrichment of target sequences compared to the whole genomic background. It can be interpreted as the proportion of sequences captured versus non-captured, regardless of the specificity.
Capture sensitivity: Fraction of captured targeted sequences (detected true positives) over the total targeted sequences present in the sample (total true positives= true positives + false negatives).
Capture specificity: Fraction of non-targeted sequences not captured (true negatives) over the total non-targeted sequences (true negatives + false positives). Maximum specificity is reached when all sequences that are captured represent target sequences (no “by-catch”).
Coverage: The number of sequencing reads covering a specific site on the genome. Read coverage per nucleotide is usually expressed as an average across a given genetic region or sample. This term is usually used synonymous to the term sequencing depth.
*De novo* assembly: Refers to a reference-free assembly approach producing contig sequences. In this approach contigs are being constructed from overlapping sequencing reads usually by applying a graph theory approach.
Deaminated DNA: Strands of DNA that have lost bases or where the bases have been transformed by deaminases or by spontaneous deamination (e.g. when a cytosine is transformed into a uracil). Deamination can cause G+C pairs to transform into A+T pairs, usually as a result of DNA damage and degradation.
Horizontal gene transfer: Transfer of genetic material between organisms or genomes that does not occur from parent to offspring (vertical transfer). Common among Bacteria and Archaea.
Hybridization rate: The fraction of fragments that hybridize to baits (related to capture efficiency).
Molecular inversion probe amplification: Sequence capture technique that uses as baits two primers that are complement to the 5′ and 3′ ends of the targeted segment (linker region). This design is such that the targeted region remains in the gap between the hybridized primer ends of the bait. After hybridization, during which the bait undergoes circularization, a DNA polymerase fills the gap between primers with the sequence complementary to the targeted region. Other target sequence capture methods differ from molecular inversion probe amplification by designing baits to bind to the targeted sequence itself.
PHRED quality score: A measurement of the quality of identification assigned to every sequenced nucleotide, i.e. how confident is the identification of each nucleotide during sequencing. The PHRED scores are encoded in ASCII characters in the line below every sequence in a fastq file.
Phylogenetically informative characters: DNA features, such as single-nucleotide variants or length polymorphisms, which are polymorphic in a dataset and inform on phylogenetic relationships.
RAD-seq: Restriction site associated DNA sequencing methods allow for the sequencing of random DNA fragments cut at specific sites using restriction enzymes (e.g. Emerson et al., 2010). Unlike target sequence capture, the regions obtained by RAD-seq are not pre-selected and focusing coverage over selected regions is, in principle, not possible.
Sequencing library: A collection of DNA fragments in solution, usually size-selected, that has been modified with synthetic DNA adapters to prepare for sequencing on a High throughput sequencing platform. The DNA in a library can originate from an individual sample, a pool of individuals, or an environmental sample.
Target sequence capture: The targeted enrichment of specific genomic regions prior to sequencing.
Touch-down temperature array: It is an approach used to increase amplification and/or hybridization efficiency without compromising specificity. Higher annealing temperatures increase specificity but reduce efficiency. By programing the thermocycler to decrease the annealing temperature at regular intervals every cycle, specific amplification or hybridization is ensured during the early cycles while increasing efficiency at the latest cycles.
Transcriptome: The collection of all RNA molecules in an organism or particular cell type.
Ultraconserved Elements (UCE): Conserved regions on the genome that show very few or no nucleotide substitutions, deletions, or insertions, when compared across deeply divergent taxa, while being flanked by more variable regions. These regions represent suitable targets for baits that can be applied across large phylogenetic scales.
Whole-transcriptome shotgun sequencing (WTSS): Also known as RNA-sequencing. It refers to the sequencing of randomly fragmented cDNA obtained from extracted RNA via reverse-transcription. WTSS enables the sequencing of coding mRNA, snRNA, and non-coding RNA.
